# Pre-contrast computed tomography reveals distinct characteristics and anatomical distribution of presumably lymphomatous lymph nodes in canine multicentric lymphoma

**DOI:** 10.14202/vetworld.2026.1521-1532

**Published:** 2026-04-24

**Authors:** Somchin Sutthigran, Kongthit Horoongruang, Supasek Sarachitti, Chutimon Thanaboonnipat, Anudep Rungsipipat, Nan Choisunirachon

**Affiliations:** 1 Department of Surgery, Faculty of Veterinary Science, Chulalongkorn University, Bangkok 10330, Thailand; 2 Small Animal Hospital, Faculty of Veterinary Science, Chulalongkorn University, Henri Dunant Rd., Pathumwan, Bangkok 10330, Thailand; 3 Crystal Pet CT Scan Center, Crystal Pet Hospital, Bangkhen, Bangkok 10220, Thailand; 4 Center of Excellence for Companion Animal Cancer, Department of Veterinary Pathology, Faculty of Veterinary Science, Chulalongkorn University, Henri Dunant Rd., Pathumwan, Bangkok10330, Thailand

**Keywords:** computed tomography, dog, Hounsfield unit, lymph node, lymphoma, multicentric lymphoma, pre-contrast CT, veterinary oncology

## Abstract

**Background and Aim:**

Canine multicentric lymphoma (CML) is one of the most common hematopoietic neoplasms in dogs and is characterized by generalized lymphadenopathy involving peripheral and visceral lymph nodes. Accurate identification of lymphomatous lymph nodes (LLNs) is essential for diagnosis, staging, and monitoring treatment response. Conventional diagnostic methods such as palpation, radiography, and ultrasound may be limited by anatomical superimposition, restricted visualization of deep structures, and operator dependency. Computed tomography (CT) provides high-resolution, whole-body imaging and may allow more reliable differentiation between abnormal and normal lymph nodes. However, information regarding pre-contrast CT characteristics of LLNs in dogs remains limited. This study aimed to describe the anatomical distribution of presumably lymphomatous lymph nodes and to evaluate pre-contrast CT parameters, including size, morphology, and attenuation values, for distinguishing LLNs from normal lymph nodes (NLNs).

**Materials and Methods:**

This retrospective, multicenter observational study evaluated medical records and pre-contrast CT images of dogs examined between February 2014 and February 2024 at two veterinary diagnostic centers in Bangkok, Thailand. The study included two groups: dogs diagnosed with CML confirmed by cytologic or histopathologic examination and dogs without lymph node-related diseases. A total of 99 presumably LLNs from nine dogs with CML and 138 NLNs from nine control dogs were analyzed. CT parameters assessed included lymph node size (width, depth, length, and depth-to-length ratio), nodal appearance (homogeneity, contour, shape, and hilar fat), and CT attenuation values of lymph nodes and perinodal fat. Statistical comparisons were performed using independent t-tests, Mann–Whitney U tests, and chi-square tests. Receiver operating characteristic analysis was used to determine the optimal cutoff value for CT attenuation.

**Results:**

Peripheral lymph nodes, particularly the mandibular, superficial inguinal, and medial iliac nodes, were most frequently affected in CML. Presumably LLNs were significantly larger in width, depth, and length than NLNs (p < 0.001), but the depth-to-length ratio did not differ significantly. LLNs more commonly exhibited heterogeneous attenuation, irregular contours, and absence of hilar fat (p < 0.001). CT attenuation values of both lymph nodes and perinodal fat were significantly higher in LLNs than in NLNs. A cutoff value greater than 31.37 Hounsfield units differentiated LLNs from NLNs with 82.8% sensitivity and 70.8% specificity.

**Conclusion:**

Pre-contrast CT is a valuable diagnostic tool for identifying LLN in dogs with multicentric lymphoma. Increased nodal size, irregular morphology, absence of hilar fat, and higher CT attenuation values, particularly a cutoff value above 31.37 Hounsfield units, can assist in differentiating abnormal from normal lymph nodes. These findings may improve diagnostic accuracy, guide biopsy site selection, and support monitoring of treatment response in canine lymphoma.

## INTRODUCTION

Lymphoma is a common hematopoietic neoplasm in dogs, accounting for approximately 7%–24% of all canine tumours and 83% of all hematopoietic neoplasms [1]. The incidence rate ranges from 19.9 to 107 cases per 100,000 dogs per year [2, 3]. More than 80% of cases present as canine multicentric lymphoma (CML), characterized by generalized lymph node enlargement [1, 4]. Although cytological or histopathological examinations remain the gold standard for diagnosis, treatment response is currently evaluated according to the Veterinary Cooperative Oncology Group (VCOG) guidelines, which involve measuring the size of lymphomatous lymph nodes (LLNs) [5]. However, this method is insufficient for accurately determining remission status [6].

Diagnostic imaging techniques, including radiography and ultrasonography (US), are crucial for diagnosing and monitoring CML [7–10]. Thoracic and abdominal radiography can detect abnormalities in more than 70% of dogs with CML and can be used to assess organ involvement during staging. However, this method is limited by nonspecific findings and anatomical superimposition [7, 10]. Abdominal US, including B-mode and elastography, can differentiate between benign and malignant lymph nodes, particularly LLNs [8, 9, 11]. Nevertheless, US evaluation may be obscured by gastrointestinal gas or ingesta, resulting in incomplete assessment. These limitations may lead to underestimation of disease extent [12, 13].

Computed tomography (CT) is an advanced imaging modality that has become increasingly used in veterinary medicine because it provides higher spatial resolution and eliminates anatomical superimposition, making it more effective than conventional imaging methods such as radiography and US [14].

Despite the increasing use of CT in veterinary oncology, most previous studies have primarily focused on its diagnostic ability for detecting lymph node enlargement, organ involvement, and metastatic lesions in dogs with lymphoma, rather than its application for treatment monitoring and remission assessment [15–19]. Recent reports have indicated that CT may provide more precise evaluation of lymph node size and internal architecture than conventional imaging modalities and may improve the accuracy of staging and follow-up examinations [20, 21]. However, standardized criteria for the use of CT in monitoring therapeutic response in dogs with CML have not yet been clearly established. In particular, limited information is available regarding the comparison between CT findings and the currently recommended VCOG response evaluation method based on LLN measurements. Consequently, the clinical usefulness of CT for determining remission status and detecting residual disease in dogs with CML remains unclear.

Therefore, the present study aimed to evaluate the usefulness of CT for assessing treatment response in dogs with CML and to compare CT findings with the response evaluation criteria recommended by VCOG. This study investigated the ability of CT to detect changes in LLNs and internal organ involvement during and after chemotherapy and to determine whether CT provides additional information for remission assessment compared with conventional measurement-based evaluation. The results of this study are expected to clarify the clinical role of CT in monitoring CML and to provide evidence for improving imaging-based criteria for treatment response in canine lymphoma.

## MATERIALS AND METHODS

### Ethical approval

Because this study was retrospective and used archived clinical data obtained during routine diagnostic procedures, no animals were subjected to additional experimental handling, treatment, or imaging for research purposes. All CT examinations and clinical procedures had been performed as part of standard veterinary care according to institutional guidelines. Therefore, the requirement for additional animal experimentation approval was waived by the respective ethics committees.

Written informed consent for diagnostic procedures and the use of medical records for research and academic purposes was obtained from the owners at the time of admission to the hospital. All data were anonymized before analysis to protect the identity of the animals and their owners.

The study was conducted in accordance with institutional policies for the use of clinical data in research and complied with national guidelines for animal welfare and veterinary clinical practice.

### Study period and location

This retrospective, multicenter observational study evaluated medical records and pre-contrast CT images of dogs collected between February 2014 and February 2024. The study was conducted at two veterinary diagnostic centers: the Diagnostic Imaging Unit, The Small Animal Hospital, Faculty of Veterinary Science, Chulalongkorn University, Bangkok, Thailand, and the Crystal Pet CT Scan Center, Crystal Pet Hospital, Bangkok, Thailand. The study protocol was approved by the Institutional Review Boards of both centers.

### Study population and case selection

Medical records from the hospital information systems of the two centers were searched using the keywords “multicentric lymphoma” or “lymphoma with multicentric form.” Records were included when the diagnosis of CML was confirmed by cytologic or histopathologic examination and whole-body CT imaging had been performed. Records were excluded if the diagnosis of lymphoma was incomplete based on cytologic or histopathologic evaluation, or if whole-body CT imaging was not available.

All dogs were positioned in sternal recumbency under general anesthesia with respiratory control. Pre-contrast whole-body CT images were obtained from the head to the pelvic limbs. Scans were performed using a 64-slice helical CT scanner (Optima CT660; GE Healthcare, Tokyo, Japan) at the Diagnostic Imaging Unit or a 128-slice helical CT scanner (NeuViz; Neusoft Medical Systems, Liaoning, China) at the Crystal Pet CT Scan Center. For both systems, acquisition parameters were standardized to ensure consistency. Tube voltage was set at 120 kVp, tube current at 200 mAs, and pitch ranged from 0.9 to 1.1. Slice thickness ranged from 1.00 to 1.25 mm depending on body size. Images were reconstructed using a standard soft tissue kernel with a 512 × 512 matrix.

Pre-contrast whole-body CT images covering the region from the head to the thoracic and pelvic limbs were included and classified into two groups: (i) CML group, consisting of dogs with multiple lymph node enlargements confirmed by cytology or histopathology; (ii) no apparent lymph node-related disease group, consisting of dogs without diseases affecting primary drainage lymph nodes, including infectious, inflammatory, or neoplastic conditions that could cause lymph node enlargement, metastasis, or systemic inflammatory changes.

Additional exclusion criteria included incomplete whole-body imaging, lack of cytologic or histopathologic confirmation of CML, other anatomical forms of lymphoma, prior treatment with chemotherapy or corticosteroids, presence of abnormal organs that could affect lymph nodes (for example, periodontal disease or other neoplastic conditions), and poor-quality images caused by motion artifacts or slice thickness greater than 1.25 mm.

Clinical and laboratory information was obtained from the hospital information systems, including age, breed, sex (with neuter status), and BW.

### CT image analysis

All CT images were retrieved from the Picture Archiving and Communication System in Digital Imaging and Communications in Medicine format. Images were re-evaluated using DICOM viewer software (OsiriX, Geneva, Switzerland) on a non-CT workstation with a 2560 × 1440-pixel monitor. A soft tissue window setting with a width of 350 HU and a level of 40 HU was used to identify lymph nodes.

Lymphocenters were identified according to their drainage regions, including head and neck (mandibular, medial retropharyngeal, and superficial cervical lymph nodes), thoracic limb (axillary lymph center including axillary and accessory axillary lymph nodes), thoracic region (sternal, cranial mediastinal, and tracheobronchial lymph nodes), abdominal region (celiac, cranial mesenteric, caudal mesenteric, gastric, hepatic, splenic, medial iliac, internal iliac, and sacral lymph nodes), and pelvic limb (popliteal and superficial inguinal lymph nodes) [22].

Presumed LLNs in the CML group were classified based on changes in size and morphology, including irregular margins or absence of hilar fat. Normal lymph nodes (NLNs) in the no apparent lymph node-related disease group were identified based on normal appearance, as evaluated by a radiologist with 10 years of experience who was blinded to the clinical diagnosis.

**Figure 1:**
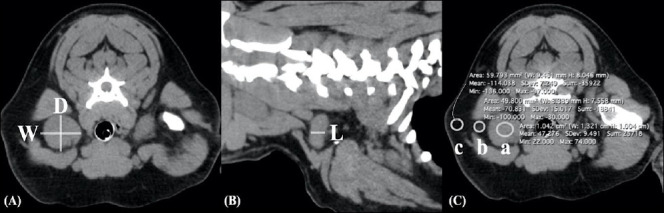
(A) Transverse computed tomography (CT) image of the right superficial cervical lymphomatous node showing width (W) measured in the lateromedial direction and depth (D) measured in the dorsoventral direction. (B) Sagittal CT image of the same node demonstrating length (L) measured in the craniocaudal direction. (C) Soft tissue window CT image (window width = 350 Hounsfield units [HU]; window level = 40 HU) showing placement of regions of interest for attenuation measurement: right axillary node (circle a), perinodal fat (circle b), and reference fat (circle c).

For each lymph node identified on CT images, the following parameters were recorded for both groups:(i) maximum width and depth measured on the transverse plane, and length measured on the sagittal plane using a single slice; the depth-to-length (D/L) ratio was then calculated (Figure 1A–B);(ii) nodal characteristics, including homogeneity (homogeneous or heterogeneous attenuation), contour (regular or irregular), shape (oval or fusiform), and hilar fat (presence or absence of a hypoattenuating center); (iii) CT attenuation values of the lymph nodes and perinodal fat.

For group comparison, regions of interest were drawn on lymph node parenchyma, perinodal fat, and reference fat. Region size was adjusted according to node size and carefully positioned to avoid hilar fat. Reference fat was defined as normal adipose tissue on the same CT slice but located away from the evaluated node and was used as an internal control for attenuation comparison (Figure 1C).

### Statistical analysis

Statistical analyses were performed using SPSS Statistics for macOS, version 29 (IBM Corp., New York, NY, USA). Descriptive statistics were used to summarize clinical data. Continuous variables, including lymph node size and CT attenuation, were assessed for normality using the Shapiro–Wilk test. Normally distributed data are presented as mean ± standard deviation, whereas non-normally distributed data are presented as median and range.

Categorical variables, including number, location, and characteristics of lymph nodes, are presented as frequencies and percentages. Continuous variables such as age, BW, lymph node size (width, depth, length, and D/L ratio), and CT attenuation values were compared between groups. The independent samples t-test was used for normally distributed data, and the Mann–Whitney U test was used for non-normally distributed data. Categorical variables were compared using the chi-squared test.

Receiver operating characteristic curve and area under the curve analyses were performed to determine optimal cutoff values of CT attenuation for differentiating presumed LLNs from NLNs. Statistical significance was set at p < 0.05.

## RESULTS

### Clinical and demographic data

Nine dogs diagnosed with CML were included in the CML group. The breeds represented were French Bulldog (n = 2) and Golden Retriever (n = 2), with one dog each of American Pit Bull, Beagle, Chihuahua, Maltese, and Poodle. In the no apparent lymph node-related diseases group, the dogs included two mixed-breed dogs and one each of French Bulldog, Golden Retriever, Japanese Akita, Maltese, Mastiff, Shih Tzu, and Siberian Husky. The primary reason for undergoing CT in the no apparent lymph node-related diseases group was evaluation of vertebral abnormalities, such as spondylosis deformans or intervertebral disc disease. The clinical data of the enrolled dogs are summarized in Table 1.

**Table 1:** Clinical and demographic characteristics of dogs in the canine multicentric lymphoma (CML) group and the no apparent lymph node-related disease group.

Characteristics	CML (n = 9)	No apparent (n = 9)
Age (years)	7.68 ± 1.62	7.89 ± 1.27
Gender		
Male	5 (55.6%)	4 (44.4%)
Female	4 (44.4%)	5 (55.6%)
Body weight (kg)	19.99 ± 5.33	19.11 ± 5.33

Among the 18 enrolled dogs, a total of 228 lymph nodes were identified in 9 dogs in the CML group. Of these, 36 nodes were definitively confirmed as LLNs by cytologic examination, whereas the remaining 94 nodes were classified as presumed LLNs based on morphological alterations in size and appearance. In the no apparent lymph node-related diseases group, 193 NLNs were identified in 9 dogs. The locations, numbers, and frequencies of the lymph nodes are presented in Table 2.

**Table 2:** Anatomical location, number, and frequency distribution of detected lymph nodes in dogs with canine multicentric lymphoma (CML) and dogs with no apparent lymph node-related disease.

Lymph nodes	CML (n = 228)		No apparent (n = 193)	

	n	% Distribution	n %	Distribution
Head and neck region				
Axillary				
Detect	18		18	9.33%
cLLNs	11 (61.11%)	8.46%		
Mandibular				
Detect	36		34	17.62%
cLLNs	22 (61.11%)	16.92%		
Medial retropharyngeal				
Detect	18		18	9.33%
cLLNs	10 (55.56%)	7.69%		
Parotid				
Detect	1		0	0.00%
cLLNs	1 (100.00%)	0.77%		
Thoracic limb region				
Superficial cervical				
Detect	18		18	9.33%
cLLNs	14 (77.78%)	10.77%		
Thoracic region				
Cranial mediastinal				
Detect	8		7	3.63%
cLLNs	2 (25.00%)	1.54%		
Sternal				
Detect	14		8	4.15%
cLLNs	4 (28.57%)	3.08%		
Tracheobronchial				
Detect	1		0	0.00%
cLLNs	1 (100.00%)	0.77%		
Abdominal region				
Gastric				
Detect	7		6	3.11%
cLLNs	2 (28.57%)	1.54%		
Hepatic				
Detect	16		14	7.25%
cLLNs	11 (68.75%)	8.46%		
Medial iliac				
Detect	18		18	9.33%
cLLNs	15 (83.33%)	11.54%		
Mesenteric				
Detect	24		9	4.66%
cLLNs	12 (50.00%)	9.23%		
Renal				
Detect	1		0	0.00%
cLLNs	1 (100.00%)	0.77%		
Splenic				
Detect	4		7	3.63%
cLLNs	3 (75.00%)	2.31%		
Pelvic limb region				
Popliteal				
Detect	18		18	9.33%
cLLNs	5 (27.78%)	3.85%		
Superficial inguinal				
Detect	18		18	9.33%
cLLNs	16 (88.89%)	12.31%		

A total of 228 lymph nodes were detected in the CML group, of which 130 were classified as comparable lymphomatous lymph nodes (cLLNs). In the no apparent disease group, 193 normal lymph nodes (NLNs) were identified.

### Comparison of pre-contrast CT parameters between presumed LLNs and NLNs

For comparison, 99 of 138 presumed LLNs and 138 of 193 NLNs were selected based on detection frequency to ensure data homogeneity by including anatomically comparable nodes from the same locations. The selected lymph nodes included axillary, hepatic, mandibular, medial iliac, medial retropharyngeal, superficial cervical, and superficial inguinal lymph nodes.

Presumed LLNs showed significantly greater width, depth, and length than NLNs (p < 0.001). However, no significant difference was observed in the D/L ratio between the two groups (*p* = 0.705). The mean ± SD values of pre-contrast CT parameters for presumed LLNs and NLNs are shown in Table 3.

**Table 3:** Mean ± standard deviation of pre-contrast CT measurements (width, depth, length, and depth-to-length ratio) of lymphomatous lymph nodes (LLNs) and normal lymph nodes (NLNs).

Parameter	LLNs (n = 99)	NLNs (n = 138)	p-value
Width (cm)	1.681 ± 0.817	0.622 ± 0.291	< 0.01
Depth (cm)	1.712 ± 0.908	0.741 ± 0.427	< 0.01
Length (cm)	2.292 ± 0.148	1.028 ± 0.575	< 0.01
D/L ratio	0.881 ± 0.544	0.998 ± 0.937	0.117

### Comparison of pre-contrast CT appearance between presumed LLNs and NLNs

**Figure 2:**
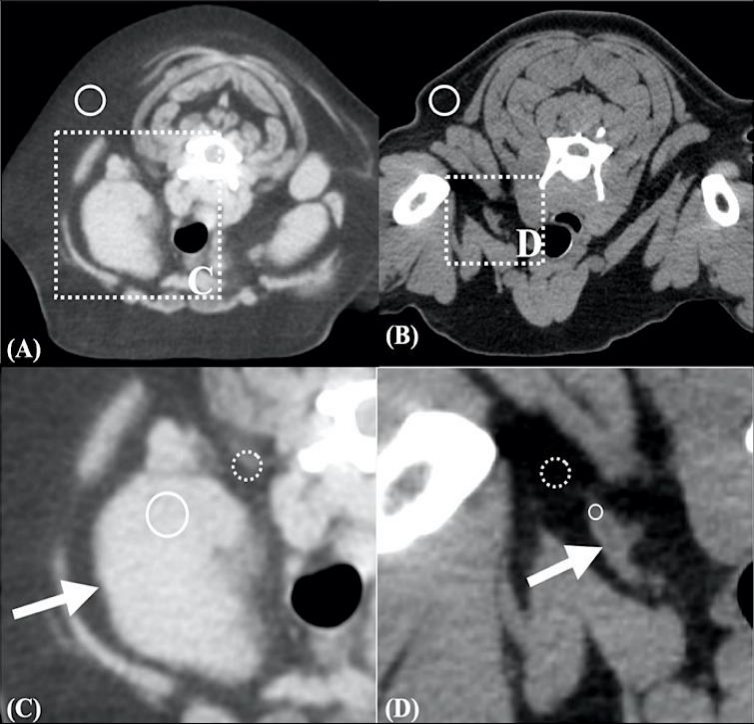
(A) Transverse CT image of a presumably lymphomatous node showing heterogeneous attenuation, irregular contours, round shape, and absence of nodal hilum fat. (B) Transverse CT image of a normal lymph node exhibiting homogeneous attenuation, regular contours, fusiform shape, and presence of nodal hilum fat appearing as a hypoattenuating central region (arrow). (C) Soft tissue window CT image (window width = 350 Hounsfield units [HU]; window level = 40 HU) of the lymphomatous node demonstrating hyperattenuating perinodal fat (arrows). (D) Soft tissue window CT image of the normal lymph node. The solid circles indicate regions of interest for the lymph node and reference fat; the dotted circles indicate perinodal fat.

The pre-contrast CT appearance of presumed LLNs differed significantly from that of NLNs. Presumed LLNs more frequently showed heterogeneous attenuation, irregular contours, and absence of hilar fat (p < 0.001) (Figure 2). However, no significant difference in lymph node shape was observed between the CML and no apparent lymph node-related diseases groups. The number and frequency of nodal characteristics in both groups are presented in Table 4.

**Table 4:** Frequency of CT features in 99 lymphomatous lymph nodes (LLNs) versus 138 normal lymph nodes (NLNs).

Nodal appearance	LLNs (n = 99)	NLNs (n = 138)	p-value
Homogeneity			< 0.001
Homogeneous	94 (94.9%)	138 (100.0%)	
Heterogeneous	5 (5.1%)	0 (0.0%)	
Contour			< 0.001
Regular	78 (78.8%)	138 (100.0%)	
Irregular	21 (21.2%)	0 (0.0%)	
Shape			0.554
Oval	64 (64.6%)	84 (60.9%)	
Fusiform	35 (35.4%)	54 (39.1%)	
Hilus fat			< 0.001
Present	2 (2.0%)	112 (81.2%)	
Absent	97 (98.0%)	26 (18.8%)	

### Comparison of CT attenuation values of lymph nodes and perinodal fat between presumed LLNs and NLNs

The pre-contrast CT attenuation values of lymph nodes in presumed LLNs were significantly higher than those in NLNs (p < 0.001). In addition, the CT attenuation values of perinodal fat in the CML group were significantly higher than those in the no apparent lymph node-related diseases group (*p* = 0.027). However, no significant difference was observed in the CT attenuation values of reference fat between the two groups (*p* = 0.991).

The mean ± SD values of CT attenuation for lymph nodes, perinodal fat, and reference fat are presented in Table 5.

**Table 5:** Mean ± standard deviation of computed tomography (CT) attenuation values (Hounsfield units [HU]) for lymph nodes, perinodal fat, and reference fat in dogs with canine multicentric lymphoma (CML) versus dogs with no apparent lymph node-related disease.

CT attenuation value (HU)	CML group	No apparent lymphadenopathy	p-value
Lymph nodes	42.036 ± 9.764	25.126 ± 13.292	< 0.001
Perinodal fat	–92.873 ± 22.093	–99.014 ± 26.481	0.027
Reference fat	–114.675 ± 13.175	–114.510 ± 15.163	0.991

### Determination of optimal cutoff value of CT attenuation for differentiating presumed LLNs and NLNs

**Figure 3:**
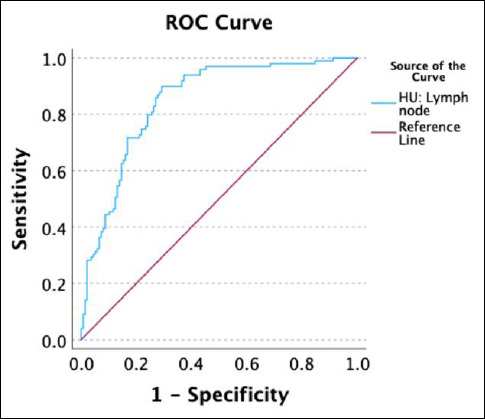
The receiver operating characteristic curves illustrate the performance of computed tomography (CT) attenuation values in distinguishing presumably lymphomatous lymph nodes (LLNs) from normal lymph nodes (NLNs). The optimal cutoff value was identified as greater than 31.37 Hounsfield units (HU). The curve demonstrates a sensitivity of 82.8% and a specificity of 70.8%, indicating the effectiveness of CT attenuation values in this differentiation.

Receiver operating characteristic curve and area under the curve (AUC) analysis determined that the optimal cutoff value of CT attenuation for identifying presumed LLNs was > 31.37 HU, with an AUC of 0.846 (95% CI 0.796–0.896), sensitivity of 82.80% (95% CI 70.99–94.32%), and specificity of 70.80% (95% CI 62.69–78.42%) (Figure 3).

## DISCUSSION

### Overall CT characteristics of lymph nodes in CML

This study demonstrated significant differences in lymph node characteristics between presumed LLNs and NLNs on CT images. Compared with NLNs, presumed LLNs were more frequently enlarged, showed irregular contours, and lacked hilar fat. In addition, CT attenuation values were significantly higher in both the lymph nodes and the surrounding perinodal fat. The present findings indicate that increased perinodal fat attenuation may represent an additional useful feature for identifying presumed LLNs. Furthermore, this study provides the first systematic whole-body mapping of pre-contrast CT attenuation in dogs with CML. The mandibular lymph nodes were the most frequently affected in the head and neck region (16.92%), followed by the superficial inguinal lymph nodes (12.31%) in the pelvic limb and the medial iliac lymph nodes (11.54%) in the abdominal region. These findings may assist in differentiating presumed LLNs from NLNs on pre-contrast CT images and may help overcome limitations of conventional diagnostic methods, including radiography and US, such as superimposition, limited resolution, and restricted visualization of deep anatomical structures.

### Distribution pattern of affected lymph nodes in CML

CML is characterized by generalized lymphadenopathy involving both peripheral and visceral lymph nodes, although the distribution is not uniform [20, 21, 23, 24]. In the present study, peripheral lymph nodes, particularly the mandibular and superficial inguinal lymph nodes, were most commonly affected, which is consistent with previous reports [20, 23]. These lymph nodes are easily detected by palpation, and changes in their size are commonly used to evaluate the response to chemotherapy [5]. However, because this disease affects both peripheral and visceral lymph nodes, evaluation based only on peripheral lymph nodes may be insufficient for accurate diagnosis and monitoring of treatment response [6]. The present results showed that medial iliac, hepatic, and mesenteric lymph nodes were most frequently affected in the abdominal region, whereas the sternal lymph nodes were most commonly involved in the thoracic region. These findings are consistent with previous observations [20]. Peripheral lymph nodes are frequently involved in lymphoma because they act as the first line of defense against pathogens and foreign antigens. Located near entry points such as the skin or oral cavity, these nodes are repeatedly exposed to antigenic stimulation, which may increase the risk of lymphoproliferative disorders, including lymphoma [25, 26].

### Detectability of lymph nodes on CT compared with normal dogs

The number of detectable lymph nodes was higher in the CML group than in the no apparent lymph node-related disease group. This is likely because LLNs are enlarged and more easily visualized on pre-contrast CT images, particularly gastric, mesenteric, and sternal lymph nodes, which are often difficult to identify in healthy dogs due to their small size or surrounding tissues. Enlargement increases their visibility on CT images [17, 27]. In addition, post-contrast CT images were not included in this study, which may have further improved the detection of small lymph nodes. Previous studies have reported that post-contrast CT imaging enhances the visibility of small lymph nodes [17, 20].

### Limitations of conventional imaging methods

Conventional imaging techniques, including radiography and US, remain essential for diagnosis and staging of CML [9, 28, 29]. Radiography can detect thoracic lymphadenopathy, pulmonary infiltration, hepatospleno-megaly, and abdominal lymphadenopathy [7, 29]. However, radiographic findings are often nonspecific, and superimposition of anatomical structures and limited contrast resolution may reduce diagnostic accuracy [20, 30]. US provides improved soft tissue contrast, absence of superimposition, and real-time evaluation [31]. Techniques such as brightness mode, elastography, and Doppler imaging may help differentiate benign and malignant lymph nodes and detect lymphomatous involvement of the liver and spleen [8, 9, 32, 33]. Nevertheless, US has limitations, including reduced visualization of abdominal organs caused by gastrointestinal gas or ingesta, operator dependence, and incomplete evaluation of disease extent [12, 13]. In addition, intrathoracic lymph nodes, including mediastinal and tracheobronchial nodes, are difficult to assess due to reverberation artifacts caused by air-filled lungs [12, 13, 31].

### CT characteristics of NLN

CT has become an essential imaging modality in veterinary medicine because it provides high spatial resolution, rapid acquisition, whole-body evaluation, and three-dimensional reconstruction [14, 34]. It also overcomes several limitations of radiography and US, including superimposition of structures and reverberation artifacts [12, 14, 30, 34]. NLNs on CT images typically appear well defined, with smooth margins, homogeneous attenuation, and visible hilar fat. Their shape varies depending on anatomical location, and nodal size is influenced by BW [35–38]. In agreement with previous studies, NLNs in the present study showed regular margins, homogeneous attenuation, and the presence of hilar fat. The mean CT attenuation value of NLNs was 25.12 ± 1.14 HU, which is consistent with previously reported values ranging from 11.70 to 37.00 HU in the pre-contrast phase [16, 17, 35, 36].

### CT characteristics of LLN

Malignant lymph nodes generally show enlargement, irregular or poorly defined margins, heterogeneous attenuation, and absence of hilar fat on CT images [15, 27]. The present results demonstrated that presumed LLNs had greater width, depth, and length, more irregular contours, absence of hilar fat, and higher CT attenuation values in both the lymph nodes and surrounding perinodal fat compared with NLNs, which is consistent with previous reports [20, 21]. Loss of hilar fat is commonly associated with neoplastic infiltration and abnormal lymph nodes [21, 36–38]. The mean CT attenuation values of presumed LLNs were significantly higher than those of NLNs, and a cutoff value greater than 31.37 HU was identified for differentiating presumed LLNs from NLNs, with high sensitivity and moderate specificity. This finding is comparable with previous reports describing CT values of approximately 37.5 HU for malignant lymph nodes [27].

Overlap in CT attenuation values between studies may be explained by biological variation, differences in anatomical location, variability among CT systems, and differences in imaging protocols. Therefore, CT attenuation values should not be interpreted alone but should be considered together with clinical, imaging, and pathological findings. Increased attenuation in presumed LLNs is likely caused by dense neoplastic infiltration, which replaces normal hilar fat and lymphoid architecture with highly cellular tissue, resulting in increased tissue density. Increased attenuation may also occur in reactive lymphadenopathy because of inflammation or fibrosis, leading to overlap between abnormal and NLN [27].

### Perinodal fat attenuation as an additional diagnostic feature

Perinodal changes have been described in malignant lymph nodes, including increased attenuation or streaky appearance of surrounding fat [20, 21]. In the present study, perinodal fat attenuation was significantly higher in presumed LLNs, which may reflect inflammatory response, increased vascularity, or microscopic structural changes associated with neoplastic infiltration [20, 21, 39].

### Study limitations

This study had several limitations. Because of its retrospective design, post-contrast CT images were not included, as scanning protocols were not standardized, which may affect the evaluation of contrast enhancement and attenuation values. The use of two different CT systems may have introduced variability, although acquisition parameters and reconstruction settings were standardized. The interval between cytologic or histopathologic diagnosis and CT examination was not recorded, which may have influenced nodal appearance. The number of enrolled dogs was limited because CT is not routinely used for monitoring this disease, which may reduce statistical power. Breed-related differences in body composition may also influence CT measurements. Not all presumed LLNs were confirmed cytologically or histologically, and NLNs were classified based on imaging appearance, which may allow inclusion of undetected abnormalities. The study also did not evaluate other lymph node diseases, such as metastasis or reactive lymphadenopathy, and did not assess WHO stage, substage, or immunophenotype. In addition, interobserver and intraobserver agreement was not evaluated. Future studies should include a larger population, additional lymph node conditions, post-contrast imaging, and longitudinal evaluation during treatment.

## CONCLUSION

This study demonstrated that CT provides valuable quantitative and morphological information for differentiating presumed LLNs from NLNs in dogs with CML. Presumed LLNs were significantly larger, more likely to have irregular contours, absence of hilar fat, and higher CT attenuation values compared with NLNs. In addition, attenuation of the surrounding perinodal fat was significantly increased in presumed LLNs, suggesting that perinodal fat changes may serve as an additional diagnostic indicator. The most frequently affected lymph nodes in this study were the mandibular lymph nodes in the head and neck region, followed by the superficial inguinal lymph nodes in the pelvic limb and the medial iliac lymph nodes in the abdominal region. These findings confirm that both peripheral and visceral lymph nodes should be evaluated for accurate assessment of this disease.

From a clinical perspective, the results indicate that CT may overcome several limitations of conventional evaluation methods such as palpation, radiography, and US, particularly for detecting deep or non-palpable lymph nodes. The cutoff value of 31.37 HU identified in this study may assist clinicians in distinguishing abnormal lymph nodes from normal ones and may help prioritize biopsy sites or monitor treatment response in anatomically inaccessible regions. However, CT attenuation values should always be interpreted together with clinical findings, cytologic or histopathologic results, and other imaging features.

The main strength of this study is the systematic whole-body evaluation of lymph nodes using standardized pre-contrast CT images, which allowed comparison of multiple lymphocenters within the same population. This study also provides the first comprehensive mapping of pre-contrast CT attenuation patterns in dogs with CML and highlights the diagnostic value of combining nodal morphology with quantitative attenuation measurements, including perinodal fat evaluation.

Several limitations should be considered when interpreting the results. The retrospective design prevented standardization of imaging protocols, and post-contrast CT images were not included. The number of enrolled dogs was limited, and not all presumed LLNs were confirmed by cytologic or histopathologic examination. In addition, the use of two CT systems and the absence of interobserver and intraobserver agreement analysis may have influenced the measured attenuation values. The study also did not include other lymph node diseases, such as metastatic or reactive lymphadenopathy, which may show overlapping CT features.

Future studies should include a larger population, standardized imaging protocols, and post-contrast CT evaluation to better characterize enhancement patterns. Evaluation of different disease stages, immuno-phenotypes, and longitudinal changes during treatment may further improve the diagnostic value of CT. In addition, incorporation of quantitative CT parameters into automated or artificial intelligence–based analysis may help develop objective criteria for diagnosis and treatment monitoring in veterinary oncology.

In conclusion, CT is a useful imaging modality for the evaluation of lymph nodes in dogs with CML and provides additional information beyond conventional diagnostic methods. The combined assessment of lymph node morphology, attenuation values, and perinodal fat characteristics may improve detection of abnormal lymph nodes and support more accurate diagnosis, staging, and monitoring of this disease.

## DATA AVAILABILITY

The supplementary data can be made available from the corresponding author upon request.

## AUTHORS’ CONTRIBUTIONS

NC, AR, CT, and SS; Contributed to the conception and design of the study, SSa, KH and SS; Performed sample and data collection, NC, and SS; Performed data validation and statistical analysis, NC and SS; Contributed to writing the original draft and creating the graphs, CT, NC and AR; Supervised the research. All authors reviewed and provided critical comments on the manuscript and read and approved the final version.
